# Mammalian target of rapamycin inhibition attenuates myocardial ischaemia–reperfusion injury in hypertrophic heart

**DOI:** 10.1111/jcmm.13451

**Published:** 2018-01-04

**Authors:** Lei‐Lei Ma, Xin Ma, Fei‐Juan Kong, Jun‐Jie Guo, Hong‐Tao Shi, Jian‐Bing Zhu, Yun‐Zeng Zou, Jun‐Bo Ge

**Affiliations:** ^1^ Shanghai Institute of Cardiovascular Diseases Zhongshan Hospital and Institute of Biomedical Science Fudan University Shanghai China; ^2^ Department of Critical Care Medicine Zhejiang Provincial People's Hospital and People's Hospital of Hangzhou Medical College Hangzhou China; ^3^ Department of Endocrinology and Metabolism Shanghai Tenth People's Hospital Tongji University School of Medicine Shanghai China; ^4^ Department of Cardiology Affiliated Hospital of Qingdao University Qingdao China

**Keywords:** rapamycin, cardioprotection, cardiac hypertrophy, ischaemia reperfusion injury, oxidative stress

## Abstract

Pathological cardiac hypertrophy aggravated myocardial infarction and is causally related to autophagy dysfunction and increased oxidative stress. Rapamycin is an inhibitor of serine/threonine kinase mammalian target of rapamycin (mTOR) involved in the regulation of autophagy as well as oxidative/nitrative stress. Here, we demonstrated that rapamycin ameliorates myocardial ischaemia reperfusion injury by rescuing the defective cytoprotective mechanisms in hypertrophic heart. Our results showed that chronic rapamycin treatment markedly reduced the phosphorylated mTOR and ribosomal protein S6 expression, but not Akt in both normal and aortic‐banded mice. Moreover, chronic rapamycin treatment significantly mitigated TAC‐induced autophagy dysfunction demonstrated by prompted Beclin‐1 activation, elevated LC3‐II/LC3‐I ratio and increased autophagosome abundance. Most importantly, we found that MI/R‐induced myocardial injury was markedly reduced by rapamycin treatment manifested by the inhibition of myocardial apoptosis, the reduction of myocardial infarct size and the improvement of cardiac function in hypertrophic heart. Mechanically, rapamycin reduced the MI/R‐induced iNOS/gp91^phox^ protein expression and decreased the generation of NO and superoxide, as well as the cytotoxic peroxynitrite. Moreover, rapamycin significantly mitigated MI/R‐induced endoplasmic reticulum stress and mitochondrial impairment demonstrated by reduced Caspase‐12 activity, inhibited CHOP activation, decreased cytoplasmic Cyto‐C release and preserved intact mitochondria. In addition, inhibition of mTOR also enhanced the phosphorylated ERK and eNOS, and inactivated GSK3β, a pivotal downstream target of Akt and ERK signallings. Taken together, these results suggest that mTOR signalling protects against MI/R injury through autophagy induction and ERK‐mediated antioxidative and anti‐nitrative stress in mice with hypertrophic myocardium.

## Introduction

Emerging evidence has demonstrated that left ventricular hypertrophy (LVH) is an independent predictor of cardiovascular events 1 and increases the risk of acute myocardial infarction (AMI) 1. LVH is present in approximately one‐third patients with AMI and is causally related to increased morbidity and mortality following AMI 2. Moreover, experimental studies have shown that animals with LVH have expanded myocardial infarct size and are refractory to cardioprotective treatments due to defective cytoprotective mechanisms following myocardial ischaemia–reperfusion (MI/R) 3, 4. Therefore, it is urgent to develop novel therapeutic strategies to further reduce infarct size, preserve cardiac function and improve the outcome of AMI patients with LVH.

Cardiac hypertrophy is an important adaptive response to haemodynamic overload, such as pressure overload 5. In response to pressure overload, the heart initiates diverse adaptive response, including autophagy, to cope with damaged protein and organelles, aggravated oxidative stress and cell death 5. Moreover, accumulating evidence has demonstrated that autophagic response plays both beneficial and adverse roles in the development of cardiac hypertrophy 6, 7, 8. Therefore, it is reasonable to speculate that the precise manipulation of autophagy may help to develop novel therapeutic strategy to rescue LVH and LVH‐related cardiovascular injury.

The mammalian target of rapamycin (mTOR) is a serine/threonine kinase and its role in regulating autophagy, oxidative stress and cardiovascular disease has received intense attention 9. mTOR is composed of two distinct complexes, mTOR1 and mTOR2. The mTOR1 complex participates in the regulation of cellular growth, while mTOR2 participates in the control of the cytoskeleton. Previous studies have shown that chronic increased mTOR activity was related to diverse disorders, including diabetes 10, obesity 11, ageing 12, uraemic cardiomyopathy 13 and cardiac hypertrophy 5. mTOR inhibition by rapamycin (a mTOR1 inhibitor) attenuated uraemia‐induced cardiac fibrosis by reducing ROS production in rat (0.2 mg/kg/day for 4 weeks) 13 and improved the cardiac function by ameliorating oxidative stress in type 2 diabetes mice (0.25 mg/kg/day for 4 weeks) 10. In addition, rapamycin was shown to mitigate pressure overload‐induced hypertrophic responses by inducing autophagy 8 and rescuing the defective cytoprotective mechanisms in mice (2 mg/kg/day for 2 weeks) 14. In addition, mTOR inhibitor rapamycin (0.25 mg/kg, once) was shown to induce preconditioning‐like myocardial protection against ischaemia reperfusion injury in mice *in vivo* 15, 16. Moreover, Volkers *et al*. 17 demonstrated that increasing mTORC2 activation while inhibiting mTORC1 signalling reduced cardiomyocyte apoptosis and necrosis after MI. These results suggest mTOR inhibition may provide promising prospect for developing novel treatment to rescue LVH‐related cardiovascular impairment.

Therefore, this study was performed to investigate whether rapamycin may ameliorate myocardial ischaemia reperfusion injury in hypertrophic heart. Given that cardiac hypertrophy is causally related to autophagy dysfunction and increased oxidative stress 18, we sought to determine the effect of rapamycin on autophagy regulation and oxidative stress in hypertrophic myocardium.

## Materials and methods

### Animals

Male C57BL/6J mice were supplied by the Shanghai Laboratory Animal Center. All the protocols used conformed to the Guide for the Care and Use of Laboratory Animals published by the US National Institutes of Health (the 8th Edition, NRC 2011), and they were approved by the Institutional Review Board of Zhongshan Hospital at Fudan University.

### Study groups and experimental protocol

Male C57BL/6J mice aged 8 weeks were given rapamycin daily (0.25 mg/kg, IP) or vehicle (0.5% DMSO, IP) for consecutive 14 days. To determine the effects of chronic mTOR inhibition on myocardial ischaemia reperfusion in mice with or without LVH, LVH was produced in mice by TAC, and then, the mice were treated with rapamycin daily (0.25 mg/kg, IP) or vehicle (0.5% DMSO, IP) for 14 days. Myocardial ischaemia–reperfusion was produced by occluding the left coronary artery for 40 min followed by reperfusion for 24 hrs in mice after 14 days of treatment.

### Transverse aortic constriction

Mice were anaesthetized using 2% isoflurane and mechanical ventilation. The aorta between the origin of the right innominate and left common carotid arteries was constricted with a 6‐0 silk suture by tieing the aorta with a bent 27‐gauge needle, which was removed after ligation. Sham‐operated animals underwent the same procedure except that the artery was not ligated. After the surgery, the mice were housed in standard animal housing conditions for 2 weeks. Then, the diastolic left ventricular posterior wall thickness was assessed using echocardiography.

### Myocardial infarction protocol

After 2 weeks of rapamycin treatment, the surgical procedures were carried out as described previously 19. Briefly, mice were anaesthetized with 2% isoflurane and artificially ventilated. A PE‐10 tube was placed on the surface of LAD, and then, the left anterior descending artery (LAD) was ligated by an 8‐0 silk with the PE‐10 tube. After 40 min of myocardial ischaemia, the tie was removed. Epicardial cyanosis was apparent in the area at risk during 40 min of coronary artery occlusion, while successful reperfusion was confirmed by epicardial hyperaemia. Mice that fully recovered from the surgery were housed in standard animal conditions for 24 hrs.

### Immunoblotting

The expression of myocardial mTOR [Cell Signaling Technology (CST), Beverly, MA, USA; 1:1000], p‐mTOR (CST, 1:1000), S6 ribosomal protein (CST, 1:1000), p‐S6 ribosomal protein (CST, 1:1000), Akt (CST, 1:1000), p‐Akt (CST, 1:1000), LC3B (CST, 1:1000), Beclin‐1 (CST, 1:1000), p62 (CST, 1:1000), ERK1/2 (CST, 1:1000), p‐ERK1/2 (CST, 1:1000), GSK3β (CST, 1:1000), p‐GSK3β (CST, 1:1000), eNOS (Abcam, 1:1000), p‐eNOS (Abcam, 1:1000), CHOP (Abcam, 1:1000), Cyto‐C (CST, 1:1000), VDAC (CST, 1:1000), gp^91phox^ (Abcam, 1:1000), iNOS (Abcam, 1:1000) and GAPDH (CST, 1:5000) were determined by immunoblotting 20. The blot density was assessed using ImageJ 1.37 software.

### Transmission electron microscopy

The cardiac tissue was fixed with 2% glutaraldehyde for 2 hrs, then fixed in 1% OsO4 for 2 hrs and embedded in resin. The ultrathin sections were stained with uranyl acetate and lead citrate and observed under an electron microscope (Hitachi Model H‐7650, Tokyo, Japan). Random horizons were acquired by an electron microscope technician.

### Doppler echocardiography

After 24 hrs of reperfusion, the mouse was anaesthetized with 1% isoflurane. M‐mode images of left ventricular were obtained at the level of the papillary muscle tips using a Vevo 770 imaging system (VisualSonics, Toronto, Canada). The ejection fraction (EF) fractional shortening (FS) was calculated using the Teichholz formula.

### Determination of infarct size

The myocardial infarct size was assessed using 2,3,5‐triphenyltetrazolium chloride (TTC, Sigma‐Aldrich, St. Louis, MO, USA) staining after reperfusion for 24 hrs. Briefly, the coronary artery was religated, and 0.2 ml 2% Evans blue dye was injected into the right ventricular cavity to identify the unstained area as the area at risk. The hearts were harvested and frozen, sectioned into 2‐mm slices and stained in 1% TTC solution at 37°C for 10 min. The myocardial infarct size (IS) was analysed using ImageJ 1.37 software (National Institutes of Health, Bethesda, MD, USA). The myocardial infarct size was expressed as a percentage of the area at risk. The area at risk was expressed as a percentage of the left ventricular.

### Detection of myocardial apoptosis

Myocardial apoptosis was assessed by terminal deoxynucleotidyl transferase dUTP nick‐end labelling (TUNEL) staining using a fluorescein *in situ* cell death detection kit (Roche, Indianapolis, IN, USA) as we described elsewhere 20. The green fluorescein staining indicates apoptotic nuclei. TUNEL‐positive nuclei (green nuclei) were expressed as the percentage of total cell population.

### Detection of caspase activities in heart tissue

Myocardial Caspase‐3, Caspase‐8, Caspase‐9 and Caspase‐12 activity was assessed using Caspase Fluorometric Assay Kits (BioVision, Mountain View, CA, USA) according to respective instruction of the manufacturer. The activities of Caspase‐3 Caspase‐8, Caspase‐9 and Caspase‐12 were expressed as fold over the corresponding control.

### Measurement of ROS generation

Dihydroethidium (DHE) staining was used to assess *in situ* ROS levels 21. 5‐μm‐thick frozen slices without fixation were stained with DHE (5 μM) at 37°C for 30 min. The photographs were acquired using a fluorescence microscope. Fluorescent intensity was assessed using ImageJ 1.37. Myocardial superoxide production was measured by lucigenin‐enhanced chemiluminescence. The relative light units (RLU) emitted was recorded and integrated over 30‐sec. intervals for 5 min. Superoxide production was normalized with the heart weight 22.

### Determination of nitrotyrosine content in cardiac tissue

The hearts were harvested after 3 hrs of reperfusion and were cut into sections of 5 μm thick after 4% paraformaldehyde fixation. The slices were by embedded by paraffin and stained with anti‐nitrotyrosine antibody (1:100; Millipore, Billerica, MA, USA). The immunostaining was conducted by utilizing the Vectastain ABC kit (1:200, Vector Laboratories, Burlingame, CA, USA), and the images were acquired under light microscopy. The cardiac nitrotyrosine content was quantified by utilizing the Nitrotyrosine ELISA Kit (Abnova, Taiwan, China). The nitrotyrosine content was expressed as microgram/milligram of protein.

### Measurement of myocardial NO content

Myocardial NO content was assessed by determining nitrite using the Griess methods. The samples from ischaemic area were harvested after 3 hrs of reperfusion. The NO content was detected by utilizing the Total Nitric Oxide Assay Kit (Beyotime Institute of Biotechnology, Shanghai, China) following the manufacturer's instruction.

### Statistical analysis

The data are presented as the mean ± S.D. One‐way ANOVA following Newman–Keuls *post hoc* test was used for multigroup comparison. A *P* < 0.05 was considered statistically significant. All statistical analyses were performed using GraphPad Prism version 4.0 (GraphPad Prism Software, San Diego, CA, USA).

## Results

### mTOR inhibition by rapamycin attenuated aortic banding‐induced cardiac hypertrophic response

To investigate the cardiac effects of rapamycin (RAPA) treatment in aortic‐banded mice, we treated mice with rapamycin or vehicle daily for 14 days after aortic‐banded surgery. The mortality did not significantly differ between the groups (0 of six in Vehicle group, 0 of six in RAPA group, three of 12 in TAC+ Vehicle group and four of 12 in TAC+ RAPA group died within 2 weeks of the operation). To assess LV performance, echocardiography was performed to measure cardiac function after 14 days of aortic banding. As shown in Table 1, chronic pressure overload remarkably induced left ventricular hypertrophy manifested by increased diastolic posterior wall thickness, while rapamycin therapy markedly diminished diastolic posterior wall thickness (0.95 ± 0.09 in TAC+ Vehicle group *versus* 0.75 ± 0.06 in TAC+ RAPA group, *P* < 0.05, Table 1). In contrast, rapamycin treatment did not affect diastolic posterior wall thickness in normal mice without aortic banding (0.65 ± 0.05 in Vehicle group *versus* 0.63 ± 0.05 in RAPA group, *P* > 0.05, Table 1). In addition, chronic rapamycin treatment did not affect heart rate, left ventricular ejection fraction (LVEF) and left ventricular fractional shortening (LVFS) in all groups.

**Table 1:** Effects of rapamycin (RAPA) treatment on physiological measurements after transverse aortic constriction (TAC)

Group	Vehicle	RAPA	TAC+ Vehicle	TAC+ RAPA
Heart rate	485 ± 21	494 ± 24	501 ± 22	490 ± 25
Ejection fraction (%)	71.5 ± 2.8	70.4 ± 2.5	69.8 ± 2.9	69.5 ± 2.7
Fractional shortening (%)	41.1 ± 2.1	40.2 ± 1.9	39.7 ± 2.2	39.5 ± 1.8
LV posterior wall thickness (mm)				
Diastole	0.65 ± 0.05	0.63 ± 0.05	0.95 ± 0.09^a^	0.75 ± 0.06^b^
Systole	0.77 ± 0.06	0.75 ± 0.06	1.11 ± 0.10^a^	0.91 ± 0.08^b^
Body weight (g)	25.5 ± 1.2	25.2 ± 1.0	24.1 ± 1.1	23.6 ± 0.9
Heart weight (g)	102.5 ± 2.5	109.4 ± 2.8	149.2 ± 4.2^a^	129.6 ± 3.5^b^
Tibial length (mm)	16.5 ± 0.2	16.7 ± 0.2	16.6 ± 0.2	16.7 ± 0.2
Heart weight/body weight (mg/g)	4.26 ± 0.11	4.27 ± 0.10	6.15 ± 0.52^a^	5.32 ± 0.41^b^
Heart weight/tibial length (mg/mm)	6.3 ± 0.21	6.4 ± 0.16	8.5 ± 0.32^a^	7.4 ± 0.21^b^

Effects of rapamycin (RAPA) on physiological measurements after transverse aortic constriction (TAC) for 2 weeks. Vehicle refers to sham‐operated mice received DMSO; RAPA refers to sham‐operated mice received rapamycin; TAC+ Vehicle refers to transverse aortic mice received DMSO; TAC+ RAPA refers to transverse aortic mice received rapamycin. Data are presented as mean ± S.D., *n* = 6–9 hearts/group.

a *P* < 0.05 *versus* Vehicle.

b *P* < 0.05 *versus* TAC+ Vehicle.

Then, mice were killed by overdose of pentobarbital sodium (150 mg/kg, IP) after echocardiography. The body weight, heart weight and tibial length were recorded. We observed that chronic pressure overload remarkably induced left ventricular hypertrophy manifested by increased heart weight and heart weight/tibial length ratio. Rapamycin treatment hindered cardiac hypertrophic response by aortic banding demonstrated by diminished heart weight and heart weight/tibial length ratio (149.2 ± 4.2 and 8.5 ± 0.32 in TAC+ Vehicle group *versus* 129.6 ± 3.5 and 7.4 ± 0.21 in TAC+ RAPA group, *P* < 0.05, Table 1), while rapamycin treatment did not reduce heart weight or heart weight/tibial length ratio in normal mice without aortic banding manifested by similar heart weight and heart weight/tibial length ratio (102.5 ± 2.5 and 6.3 ± 0.21 in Vehicle group *versus* 109.4 ± 2.8 and 6.4 ± 0.16 in RAPA group, *P* > 0.05, Table 1). In addition, chronic rapamycin treatment did not affect heart weight or heart weight/tibial length ratio in normal mice. Taken together, these results suggest that rapamycin hinder aortic banding‐induced left ventricular hypertrophy.

### Rapamycin treatment inhibited myocardial mTOR1 signal in aortic‐banded mice

**Figure 1:**
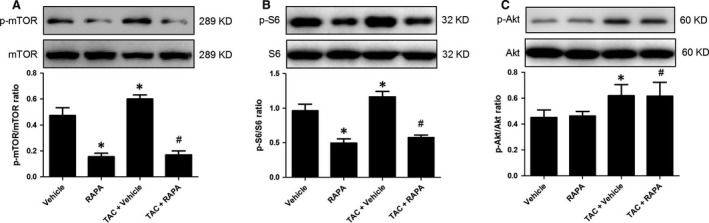
Rapamycin treatment inhibited myocardial mTOR1 signal in aortic‐banded mice. Representative immunoblots and quantitative analysis of p‐mTOR (**A**) p‐S6 ribosomal protein (**B**) and p‐Akt (**C**). *P < 0.05 *versus* Vehicle, ^#^ *P* < 0.05 *versus* TAC+ Vehicle; *n* = 6 per group.

Myocardial mTOR and S6 ribosomal protein is the downstream target of mTORC1, while the phosphorylated Akt (Ser^473^) is main target of mTORC2 23. Therefore, we assess the myocardial expression of mTOR, S6 ribosomal protein and Akt in aortic‐banded mice treated with rapamycin. Aortic banding prompted the activation of mTOR, S6 ribosomal protein and Akt demonstrated by increased phosphorylated mTOR, prompted S6 ribosomal protein phosphorylation and enhanced Akt phosphorylation (Fig. 1A–C). In contrast, rapamycin treatment inhibited pressure overload‐elicited mTOR and S6 ribosomal protein activation (Fig. 1A and B) but did not alter phosphorylated Akt (Fig. 1C) in aortic‐banded mice. Similarly, chronic administration of rapamycin inhibited myocardial mTOR and S6 ribosomal protein expression (Fig. 1A and B) but did not alter phosphorylated Akt in normal mice without aortic banding (Fig. 1C). Taken together, these findings demonstrated that rapamycin hindered overactivated mTOR1 signal in aortic‐banded mice.

### mTOR inhibition by rapamycin alleviated autophagy dysfunction in aortic‐banded mice

**Figure 2:**
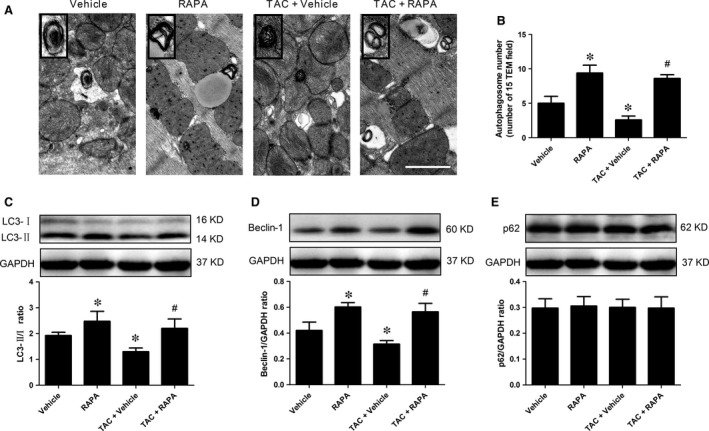
mTOR inhibition by rapamycin alleviated autophagy dysfunction in aortic‐banded mice. **A**, Representative autophagosome photographs, assessed by transmission electron microscopy, scale bar = 500 nm. **B**, The number of autophagosomes, quantified by analysis of 15 fields in each sample. **C**, The expression of LC3 in myocardial tissue, detected by immunoblotting. **D**, Beclin‐1. **E**, p62. *P < 0.05 *versus* Vehicle, ^#^ *P* < 0.05 *versus* TAC+ Vehicle; *n* = 6 per group.

The disturbance of autophagy has been demonstrated as an important contributor for pathological cardiac hypertrophy during pressure overload 18. As shown in Fig. 2A, pressure load markedly hinder autophagosome accumulation, while rapamycin prevented such alteration. To demonstrate that autophagy activity regulation was related to rapamycin‐induced cardioprotection, we assessed the conversion of the soluble form of LC3 (LC3‐I) to the cleaved autophagosome‐associated form (LC3‐II) and the Beclin‐1 activation in aortic‐banded mice. We found that the ratio of LC3‐II/LC3‐I and the Beclin‐1 were remarkably reduced by aortic banding (Fig. 2C and D), suggesting exacerbated autophagy dysfunction and decreased autophagosome accumulation. It is noteworthy that aortic banding did not significantly increase the expression of p62 protein (serving as a specific autophagic substrate protein representing autophagic flux) (Fig. 2E). In contrast, rapamycin treatment remarkably prompted the conversion of LC3‐I to LC3‐II, up‐regulated Beclin‐1 level and increased autophagosome abundance in normal and aortic‐banded mice (Fig. 2A–E). Altogether, these findings demonstrated that chronic pressure load suppressed myocardial autophagy, which could be rescued by chronic rapamycin treatment.

### mTOR inhibition by rapamycin inhibited MI/R‐induced myocardial apoptosis, infarct size and cardiac dysfunction in aortic‐banded mice

**Figure 3:**
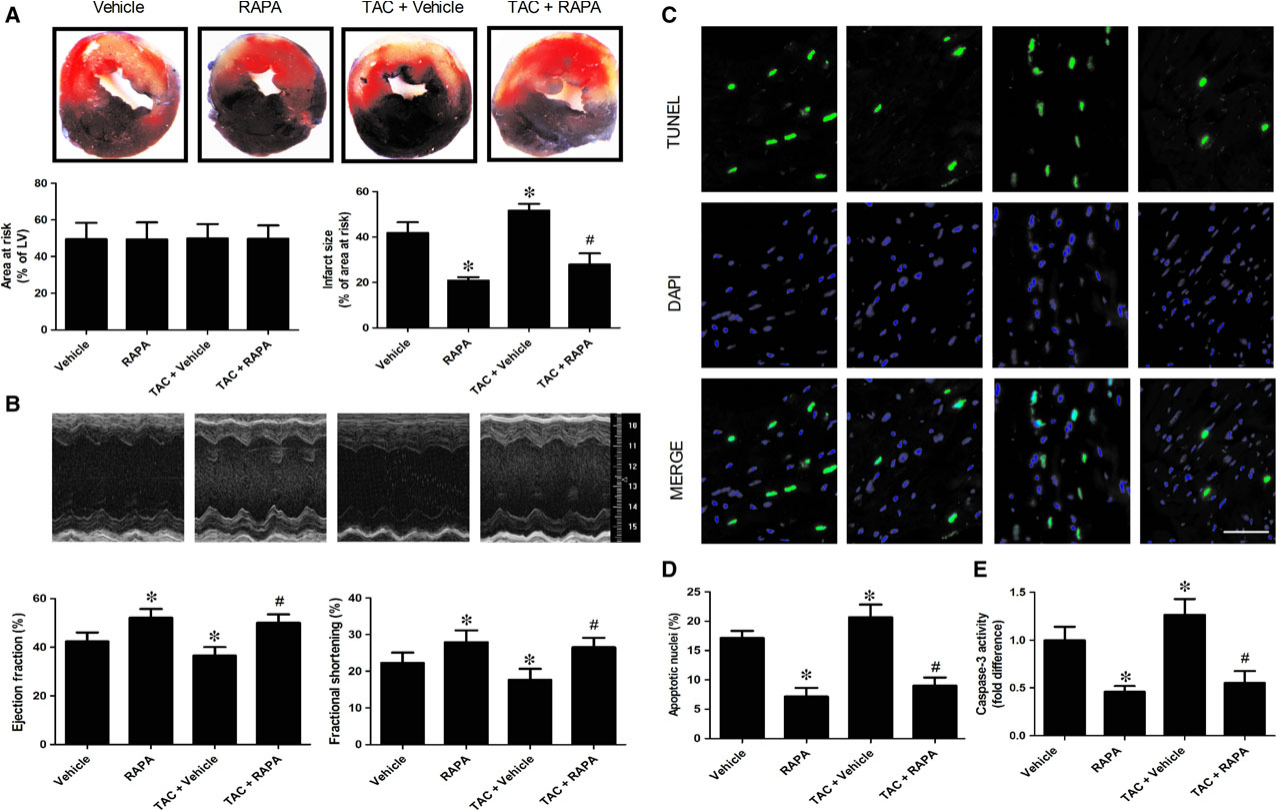
mTOR inhibition by rapamycin inhibited MI/R‐induced myocardial apoptosis, infarct size and cardiac dysfunction in aortic‐banded mice following MI/R. **A**, Myocardial infarct size, detected by Evans blue and TTC staining at 24 hrs after MI/R. **B**, Cardiac function (left ventricular ejection fraction and fractional shortening), measured using Doppler echocardiography at 24 hrs after MI/R. **C**, Representative TUNEL staining photographs after 3 hrs of MI/R, scale bar = 50 μm. **D**, The quantitative analysis of apoptotic nuclei. **E**, Myocardial Caspase‐3 activity following *in situ* MI/R. *P < 0.05 *versus* Vehicle, ^#^ *P* < 0.05 *versus* TAC+ Vehicle; *n* = 6 per group.

To determine whether chronic rapamycin treatment would render the heart resistant to subsequent ischaemic stress in aortic‐banded mice, rapamycin was administrated daily for 2 weeks in male mice, followed by subsequent exposure to regional myocardial ischaemia by *in situ* coronary artery ligation. Compared with vehicle, chronic administration of rapamycin for 14 days remarkably decreased myocardial infarct size in RAPA group (20.84% ± 1.50% in RAPA group *versus* 41.89% ± 4.65% in Vehicle group, *P* < 0.05, Fig. 3A). Especially important, rapamycin remarkably ameliorated MI/R‐induced myocardial necrosis assessed by infarct size in aortic‐banded mice (27.94% ± 4.89% in TAC+ RAPA group *versus* 51.67% ± 2.93% in TAC+ Vehicle group, *P* < 0.05, Fig. 3A), while the AAR did not significantly differ among all groups. Moreover, chronic rapamycin treatment remarkably decreased MI/R‐induced cardiomyocyte apoptosis demonstrated by reduced the number of TUNEL‐positive cell nucleus (*P* < 0.05, Fig. 3D) and inhibited Caspase‐3 activation (*P* < 0.05, Fig. 3E). To assess LV performance, echocardiography was performed to measure cardiac function after 24 hrs of reperfusion. Compared with vehicle, chronic administration of rapamycin for 14 days improved cardiac function demonstrated by enhanced LVEF and LVFS (52.12% ± 3.58% and 27.95% ± 3.24% in RAPA group *versus* 42.37% ± 3.72% and 22.29% ± 2.80% in Vehicle group, *P* < 0.05, Fig. 3B) in normal mice after MI/R. Importantly, chronic rapamycin treatment prompted cardiac functional recovery demonstrated by increased LVEF and LVFS (50.10% ± 3.41% and 26.56% ± 2.55% in TAC+ RAPA group *versus* 36.57% ± 3.50% and 17.64% ± 3.02% in TAC+ Vehicle group, *P* < 0.05, Fig. 3B) in aortic‐banded mice subjected to MI/R. Taken together, these findings indicate that rapamycin treatment rescues cardiomyocyte death and improved cardiac function during MI/R in both normal and aortic‐banded mice.

### mTOR inhibition by rapamycin suppressed the endoplasmic reticulum stress‐ and mitochondria‐mediated apoptosis pathway

**Figure 4:**
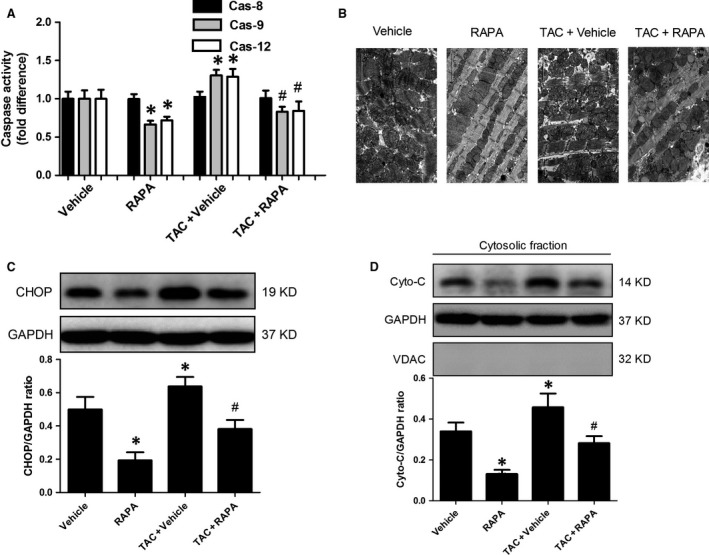
mTOR inhibition by rapamycin suppressed the endoplasmic reticulum stress‐ and mitochondria‐mediated apoptosis pathway in ischaemic/reperfused myocardium. **A**, Myocardial Caspase activity following *in situ* MI/R. **B**, Representative mitochondrial photographs, detected by transmission electron microscopy, scale bar = 2 μm. **C**,** D**, Representative immunoblots and quantitative analysis of CHOP (**C**) and Cyto‐C (**D**). *P < 0.05 *versus* Vehicle, ^#^ *P* < 0.05 *versus* TAC+ Vehicle; *n* = 6 per group.

To provide mechanistic insights into rapamycin‐induced myocardial protective effects against apoptosis, the endoplasmic reticulum stress‐ and mitochondria‐mediated apoptotic pathway was assessed. In the normal mouse heart, chronic rapamycin treatment suppressed MI/R‐elicited Caspase‐12 (a mediator of endoplasmic reticulum stress) and Caspase‐9 activation (a participator for mitochondria‐mediated apoptosis) but did not alter Caspase‐8 activity (a mediator of the death receptor signalling) (Fig. 4A). Moreover, rapamycin hindered CCAAT/enhancer‐binding protein homologous protein (CHOP) activation (Fig. 4C), suppressed Cyto‐C release (Fig. 4D) and attenuated mitochondrial damage (Fig. 4B) in normal mice followed by MI/R. Intriguingly, the aforementioned findings were also observed in aortic‐banded mice treated with rapamycin demonstrated by suppressed Caspase‐12 and Caspase‐9 activation (Fig. 4A), inhibited CHOP expression (Fig. 4C), suppressed Cyto‐C release (Fig. 4D) and preserved intact mitochondria (Fig. 4B). Taken together, these findings indicate that rapamycin ameliorated MI/R‐elicited endoplasmic reticulum stress and mitochondrial impairment and thus reduced cardiomyocyte apoptosis.

### mTOR inhibition by rapamycin attenuated oxidative stress by suppressing gp^91phox^ expression

**Figure 5:**
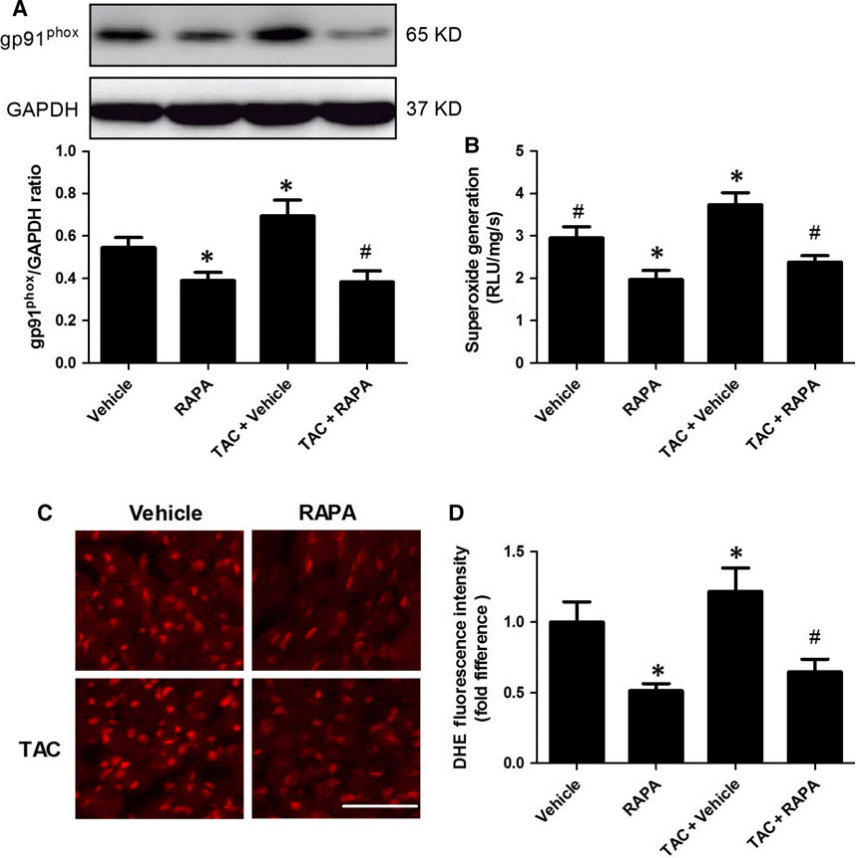
mTOR inhibition by rapamycin attenuated oxidative stress by suppressing gp^91phox^ expression in ischaemic/reperfused myocardium. **A**, The myocardial expression of gp^91phox^, determined by immunoblotting. **B**, Superoxide production in ischaemic/reperfused cardiac tissue, evaluated by lucigenin‐enhanced chemiluminescence. **C**, Myocardial superoxide at steady‐state level, measured by *in situ* dihydroethidium (DHE) staining, scale bar = 50 μm. **D**, Quantitative analyses of DHE fluorescence intensity. *P < 0.05 *versus* Vehicle, ^#^ *P* < 0.05 *versus* TAC+ Vehicle; *n* = 6 per group.

Oxidative stress has been proved as an important upstream mediator of apoptosis 24; therefore, we investigated the roles of mTOR inhibition in regulating oxidative stress to provide mechanistic insights into the cardioprotection of rapamycin against apoptosis. We found that rapamycin treatment remarkably ameliorated MI/R‐elicited superoxide production (Fig. 5B and C) and gp91^phox^ overexpression (Fig. 5A) in normal mice. Furthermore, chronic administration of rapamycin markedly alleviated myocardial oxidative stress in aortic‐banded mice manifested by reduced superoxide production (Fig. 5B and C) and gp91^phox^ overexpression (Fig. 5A). Collectively, these findings imply that rapamycin induces cardioprotection by ameliorating myocardial oxidative stress *via* down‐regulated gp^91phox^ expression.

### mTOR inhibition by rapamycin attenuated nitrative stress by suppressing iNOS expression

**Figure 6:**
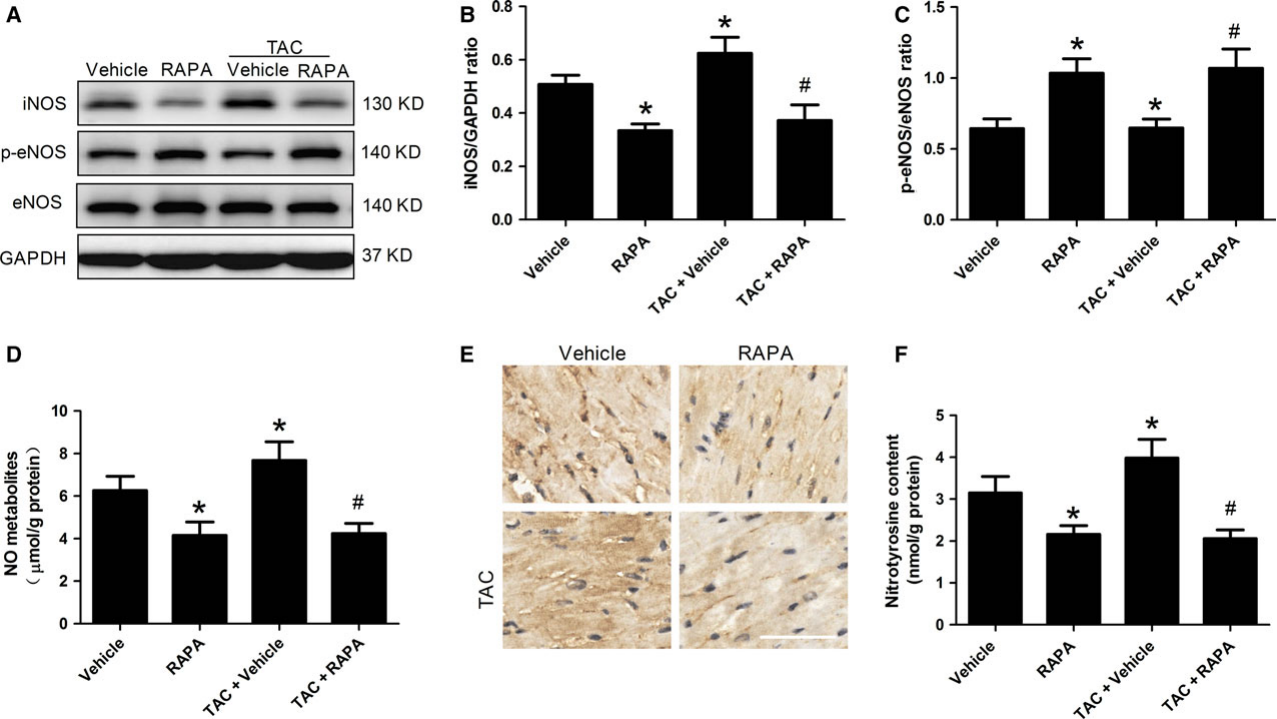
mTOR inhibition by rapamycin attenuated nitrative stress by suppressing iNOS expression in ischaemic/reperfused myocardium. **A**, Representative immunoblotting photographs for myocardial iNOS, p‐eNOS, eNOS and GAPDH expression. **B**, Quantitative analyses of myocardial iNOS expression. **C**, Myocardial p‐eNOS expression. **D**, NO content in ischaemic/reperfused myocardial tissue, measured using the Griess methods. **E**, Representative photographs for myocardial nitrotyrosine staining, scale bar = 50 μm. **F** Quantitative analyses of myocardial nitrotyrosine content. *P < 0.05 *versus* Vehicle, ^#^ *P* < 0.05 *versus* TAC+ Vehicle; *n* = 6 per group.

It is noteworthy that NO itself does not induce additional myocardial impairment under physiological conditions; however, NO interacts with superoxide and subsequently induces nitrative injury to mitochondria, protein and lipids under pathological conditions and scavenging peroxynitrite ameliorates ischaemia reperfusion injury 25. We next sought to examine whether mTOR inhibition by rapamycin could affect myocardial NO generation. Therefore, we determine myocardial total NO content and eNOS activity in both normal and aortic‐banded mice followed by MI/R. We found that the myocardial NO content was markedly reduced by rapamycin treatment, whereas the eNOS activity was significantly enhanced, as demonstrated by the reduction of myocardial NO metabolites and an increase of eNOS phosphorylation in both normal and aortic‐banded mice (Fig. 6C and D). It is accepted that increased eNOS phosphorylation would induce NO generation, which seems to be conflicting with our present finding. Nonetheless, these paradoxical results indicate that other forms of NOS are involved in the increase of myocardial NO content. Therefore, we further determine myocardial iNOS expression. Indeed, the myocardial iNOS expression as well as nitrotyrosine content was markedly reduced by chronic administration of rapamycin (Fig. 6B, E and F). These results demonstrated that mTOR inhibition alleviated nitrative stress by regulating the expression of iNOS and eNOS in both normal and aortic‐banded mice.

### mTOR inhibition by rapamycin activated pro‐survival protein kinase in ischaemic/reperfused myocardium

**Figure 7:**
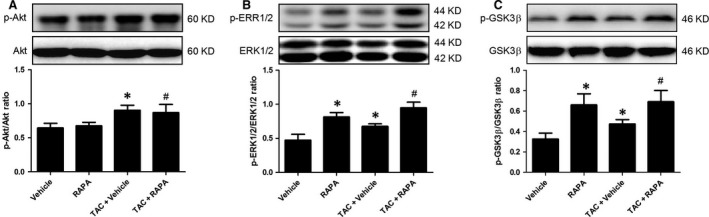
mTOR inhibition by rapamycin activated pro‐survival protein kinase in ischaemic/reperfused myocardium. Representative immunoblots and quantitative analysis of p‐Akt (**A**), p‐ERK1/2 (**B**) and p‐GSK3β expression (**C**). *P < 0.05 *versus* Vehicle, ^#^ *P* < 0.05 *versus* TAC+ Vehicle; *n* = 6 per group.

We next sought to determine the mechanism in response to eNOS phosphorylation and iNOS expression by mTOR1 inhibition. Intriguingly, the NO signalling has been shown to relate to ERK pathway 26, 27, and mTOR inhibition has been demonstrated to prompt myocardial ERK phosphorylation 15, 28; thus, we speculated that ERK may serve as a mediator to regulate NO pathway by mTOR inhibition. As shown in Fig. 7, the myocardial ERK expression was not remarkably different among all groups, whereas mTOR inhibition by rapamycin prompted ERK phosphorylation in both normal and aortic‐banded mice hearts followed by MI/R (Fig. 7B). Furthermore, ERK activation has been implicated in inactivating GSK3β, a key kinase involved in determining cardiomyocyte fate 29, and thus, we determined myocardial GSK3β expression. As expected, mTOR inhibition by rapamycin remarkably suppressed myocardial GSK3β activity manifested by up‐regulated GSK3β phosphorylation level in aortic‐banded mice (Fig. 7C). Moreover, we demonstrated that rapamycin did not affect myocardial Akt activity in aortic‐banded mice followed by MI/R (Fig. 7A). These findings imply that ERK/GSK3β signalling may play a crucial role in rapamycin‐elicited cardioprotection.

## Discussion

The salient findings from our study can be summarized as follows. First, hypertrophied myocardium is vulnerable to myocardial ischaemia reperfusion injury due to increased oxidative/nitrative stress. Second, we demonstrated for the first time that mTOR inhibition by rapamycin reduced myocardial infarct size, hindered cardiomyocyte apoptosis and preserved cardiac function in aortic‐banded mice following MI/R. Third, inhibition of myocardial mTOR signalling protects against MI/R injury through ERK‐mediated antioxidative and anti‐nitrative stress.

Although intense research efforts have been devoted to dissect the role of autophagy in cardiac hypertrophy, it remains ambiguous regarding the precise role of autophagy in cardiac hypertrophy 5. Our study demonstrated that autophagy was retarded in hypertrophic myocardium manifested by reduced LC3 expression, inhibited Beclin‐1 activation and decreased autophagosome abundance. In parallel with the present study, the induction of autophagy was documented to ameliorate hypertrophic responses while autophagy inhibition aggravates hypertrophic responses 6, 8. Nonetheless, other reports have demonstrated that autophagy induction exacerbated severe pressure overload‐induced cardiac hypertrophy and dysfunction 7, 30. These conflicting findings may be attributed to different experimental settings and intervention periods. For instance, the change in cardiac autophagic activity is time‐dependent during persistent pressure overload 31. In the acute period, pressure overload appeared to induce autophagy within 24 hrs after aortic banding. However, autophagy was retarded below the level of physiological conditions after 3 to 5 days of aortic banding, and the autophagy flux was markedly retarded at 7 days 31. Furthermore, it is well accepted that adaptive autophagy under mild cardiac hypertrophy may contribute to maintain cardiac geometry and function, whereas maladaptive autophagy under severe hypertrophic stress may accentuate cardiac hypertrophy and dysfunction. In parallel with these studies, autophagy was markedly suppressed in our cardiac hypertrophy model induced by severe pressure overload for 2 weeks, whereas chronic rapamycin treatment induced autophagy and preserved cardiac function following MI/R.

The effects of cardiac hypertrophy on MI/R have not yet been fully elucidated. Here, our study has shown markedly increased oxidative/nitrative stress in hypertrophic cardiac tissue. Furthermore, we observed increased myocardial infarct size, aggravated cardiac function and accentuated cardiomyocyte apoptosis in hypertrophic heart in comparison with that in normal ones following MI/R. Most importantly, we demonstrated for the first time that mTOR inhibition by rapamycin markedly decreased myocardial infarct size and apoptosis in aortic‐banded mice. Moreover, rapamycin treatment preserved cardiac function manifested by increased LVEF and LVFS in comparison with vehicle‐treated hypertrophic hearts. In parallel with our study, the myocardial infarct size was significantly augmented in spontaneously hypertensive rat in the setting of MI/R 3, 32. Nonetheless, other studies have shown contradictory results that hypertrophic myocardium is not markedly vulnerable to infarction during MI/R 33, 34. The reasons for these controversial findings are largely unknown but may be attributed to apparent disparity in models of cardiac hypertrophy (time of pressure overload and species) and experimental setting (myocardial ischaemic time).

Apoptosis is the major contributor for programmed cell death after MI/R injury 35. The involvement of mTOR in regulating cardiomyocyte apoptosis has been reported by several previous studies 16, 36. Nonetheless, the underlying signalling mechanisms constituting the cytoprotective effects of mTOR inhibition remain largely unknown. To provide mechanistic insights into how rapamycin exerted cardioprotection against ischaemia reperfusion injury, we investigated the effects of rapamycin on endoplasmic reticulum stress‐ and mitochondrial stress‐mediated apoptotic pathway. Our study showed chronic administration of rapamycin ameliorated MI/R‐induced endoplasmic reticulum and mitochondrial stress but did not alter Caspase‐8 activity demonstrated by inhibited CHOP increase, reduced Caspase‐12 activity, retarded cytoplasmic Cyto‐c release and preserved intact mitochondria. These findings suggest that rapamycin exerts its anti‐apoptotic effect by ameliorating endoplasmic reticulum stress and mitochondrial impairment.

MI/R‐induced DNA damage and superoxide production is the primary contributor for cardiomyocyte death and is causally related to endoplasmic reticulum stress, mitochondrial injury and cardiomyocyte apoptosis 37. Therefore, we investigated the role of mTOR on regulating oxidative/nitrative stress during MI/R. We provided first direct evidence that chronic administration of rapamycin markedly reduced gp^91phox^ activation and superoxide generation in aortic‐banded mice followed by MI/R and thus ameliorated oxidative stress‐induced myocardial impairment. It is noteworthy that NO itself does not lead to additional myocardial impairment under physiological conditions; however, NO interacts with superoxide and subsequently induces oxidative/nitrative injury to mitochondria, protein and lipids under pathological conditions 25. In addition, previous studies have proved the harmful effects of nitrative stress and scavenging peroxynitrite ameliorates ischaemia reperfusion injury 38, 39. Our present study has demonstrated that rapamycin inhibited iNOS activation, hindered NO production and reduced myocardial nitrotyrosine accumulation. Taken together, the results from our present study implicate that mTOR inhibition reduces myocardial oxidative/nitrative injury during MI/R.

mTOR inhibition is not only confined to modulate oxidative stress, but also related to cardioprotective molecules 16, 36. The present study has shown that mTOR inhibition by rapamycin markedly prompted the phosphorylated eNOS expression, and low concentration of NO produced by activated eNOS is well known to confer cardioprotective effects against necrosis and apoptosis during MI/R. Nonetheless, the role of mTOR in regulating NOS is still ambiguous. Recent studies have documented that ERK pathway is causally related to the cardioprotection by the activation of eNOS 15, 27, and ERK activation has been demonstrated to attenuate oxidative/nitrative stress 26, 40. In our murine models, selective inhibiting cardiac mTOR1 increased phosphorylated ERK expression but not Akt phosphorylation, implying that ERK signalling is involved in regulating eNOS activation by mTOR inhibition. It is accepted that iNOS signal can be regulated by a variety of stresses and transcription factors 41. The ERK signalling may also be involved in regulating iNOS activation, and Shaker *et al*. 26 demonstrated that ERK activation hindered the overproduction of iNOS in mice subjected to warm hepatic ischaemia reperfusion. Nonetheless, other molecular target downstream of mTOR may also participate in the regulation of iNOS expression by transcriptional control, and deserved further studies to clarify. Moreover, the increase of phosphorylated ERK1/2 by intermedin has been demonstrated to alleviate MI/R‐elicited ROS overload and gp^91phox^ activation 40, which provides further evidence for our findings that mTOR inhibition attenuated gp^91phox^ expression and superoxide generation through ERK1/2 signalling during MI/R.

The inactivation of GSK3β has been proved to play a crucial role in preserving myocardial survival and function during MI/R 29. We found that rapamycin prompted ERK1/2 phosphorylation to inactivate GSK3β signalling by phosphorylated GSK3β, and the phosphorylated GSK3β inhibits the opening of mitochondrial permeability transition pore (mPTP) and reduces mitochondria‐dependent apoptosis and necrosis during MI/R. However, we did not observe markedly increased Akt phosphorylation after chronic administration of rapamycin in both normal and hypertrophic heart following MI/R. These results revealed an ERK‐dependent mechanism for inhibiting mitochondria‐dependent apoptosis and necrosis. In parallel with our study, a previous study using pharmacological approach targeting inhibition of ERK signalling demonstrated that ERK signalling activation was causally related to the myocardial protective effects of acute rapamycin treatment against MI/R 15, 28. Altogether, these results indicated rapamycin inhibited MI/R and prompted cardiomyocyte survival by alleviating mitochondria‐dependent apoptosis and necrosis *via* an ERK‐dependent mechanism.

In summary, our findings reveal that mTOR signalling protects against MI/R injury through autophagy induction and ERK‐mediated antioxidative and anti‐nitrative stress in mice with hypertrophic myocardium. These findings add to our understanding of MI/R in pathological cardiac hypertrophy and may help to develop novel therapeutic strategies to further reduce infarct size, preserve cardiac function and improve the outcome of AMI patients with LVH.

## Conflict of interest

None.
